# Molecular dynamics of the immune checkpoint programmed cell death protein I, PD-1: conformational changes of the BC-loop upon binding of the ligand PD-L1 and the monoclonal antibody nivolumab

**DOI:** 10.1186/s12859-020-03904-9

**Published:** 2020-12-14

**Authors:** Bernhard Roither, Chris Oostenbrink, Wolfgang Schreiner

**Affiliations:** 1 Institute of Biosimulation and Bioinformatics, Medical University of Vienna, Spitalgasse 23/88.04.510, 1090 Vienna, Austria; 2 Institute of Molecular Modeling and Simulation, University of Natural Resources and Life Science, Vienna, Muthgasse 18, 1190 Vienna, Austria

**Keywords:** PD-1, PD-L1, Nivolumab, Molecular dynamics, BC-loop, Mathematical oncology

## Abstract

**Background:**

The immune checkpoint receptor programmed cell death protein I (PD-1) has been identified as a key target in immunotherapy. PD-1 reduces the risk of autoimmunity by inducing apoptosis in antigen-specific T cells upon interaction with programmed cell death protein ligand I (PD-L1). Various cancer types overexpress PD-L1 to evade the immune system by inducing apoptosis in tumor-specific CD8+ T cells. The clinically used blocking antibody nivolumab binds to PD-1 and inhibits the immunosuppressive interaction with PD-L1. Even though PD-1 is already used as a drug target, the exact mechanism of the receptor is still a matter of debate. For instance, it is hypothesized that the signal transduction is based on an active conformation of PD-1.

**Results:**

Here we present the results of the first molecular dynamics simulations of PD-1 with a complete extracellular domain with a focus on the role of the BC-loop of PD-1 upon binding PD-L1 or nivolumab. We could demonstrate that the BC-loop can form three conformations. Nivolumab binds to the BC-loop according to the conformational selection model whereas PD-L1 induces allosterically a conformational change of the BC-loop.

**Conclusion:**

Due to the structural differences of the BC-loop, a signal transduction based on active conformation cannot be ruled out. These findings will have an impact on drug design and will help to refine immunotherapy blocking antibodies.

## Background

Programmed cell death protein I (PD-1) is a type 1 transmembrane protein in mainly T and B cells and a so-called immune checkpoint as it promotes self-tolerance by inducing apoptosis of antigen-specific T cells, a mechanism which is often exploited by cancer cells. Thus, PD-1 has recently emerged as key target in cancer immunotherapy.

The regulatory effect of PD-1 is triggered upon binding the cell death protein I ligand 1 (PD-L1) which is mainly expressed on macrophages, dendritic cells and a variety of tissue cells [1]. Naïve T-cells reside in the lymph nodes and become activated upon interaction of the T cell receptor (TCR) with antigen-presenting cells (APC) which display antigens via the major histocompatibility complex (MHC) II. However, secondary co-stimulation of PD-1 with APC PD-L1 results in the T cell apoptosis instead [2]. The interaction between PD-1 and PD-L1 can take place in all stages of the T cell lifespan. Occurrences ranging from the early stage of T cell activation to the operational inflamed tissue site manifest the importance of the PD-1/PD-L1 pathway for the tight regulation of the immune system [3]. Cancer cells which express PD-L1 evade the host immune system by inducing apoptosis in cancer antigen specific T cells [4]. In a new approach, blocking antibodies that either target PD-1 or PD-L1 are used to disable the interaction between PD-1 and PD-L1. Thus, the T cells stay active and can target the cancer cells. Recently the PD-1 blocking antibody nivolumab was approved by the FDA. Nivolumab is clinically used for the treatment of melanoma, metastatic renal cell carcinomas, classical Hodgkin lymphoma (cHl) and non-small-cell lung carcinoma (NSCLC) [5].

Even though PD-1 is already successfully used as an immunotherapy drug target the exact mechanism of the receptor is not fully understood yet [6]. For instance, the signal transduction from the extracellular domain into the intracellular domain is still a matter of debate, enriched by modelling approaches [7–9]. Presumably, the binding of PD-L1 induces an active conformation in PD-1 which facilitates the PD-1 signaling cascade inside the T cell. Indeed, conformational changes upon ligand binding were discovered [10]. In a molecular dynamics (MD) simulation the switch from the open to closed CC’-loop conformation of PD-1 upon PD-L1 binding was described [11]. In other MD simulations the influence of mutations on ligand binding was examined [12, 13]. Also the conformational dynamics of PD-L1 were studied with MD [14, 15]. We have also performed preliminary investigations [16] which are supplemented in the current work.

In a similar approach, we examine the conformational changes of the BC-loop upon binding either PD-L1 or nivolumab. MD studies of PD-1 unbound and bound to PD-L1 and the clinically used antibody nivolumab were performed. This provided insight into flexibility of residues and conformational movements of the BC-loop which may be important for the optimization of already existing PD-1 antibodies or the design of new antibodies or small molecular compounds.

## Methods

The following notation will be used: PD-1 unbound, PD-1 – PD-L1 and PD-1 – nivolumab when the whole simulated system is addressed. PD-1_Apo,_ PD-1_PD-L1_ and PD-1_Niv_ refer specifically to PD-1 within the respective simulated system.

### Preprocessing

The crystal structures PD-1 unbound (3RRQ), PD-1 – PD-L1 (4ZQK) and PD-1 – nivolumab (5WT9) were used for simulations. With each binding partner different parts of PD-1 crystallized and therefore the files had to be manually curated first. Missing atoms and single residues were added with Swiss PDB-Viewer. The C’D-loop (residues 65–92) was taken from PD-1 – pembrolizumab (5GGS) and added to each system. The N-loop (25–34) was taken from 5WT9 and added to 3RRQ and 4ZQK. The PD-1 structures of each PDB file were aligned with VMD to orientate them in the same direction. The missing loops were copied and pasted into the PDB files. Hence, each PD-1 included the residues from 25 to 149 which represent the complete extracellular domain.

### Simulation

MD simulations were performed on a node of the Vienna Scientific Cluster (VSC) consisting of two processors (Intel Xeon E5-2650v2, 2.6 GHz, 8 cores from Ivy Bridge-EP family) and a GPU (NVIDIA Pascal GeForce GTX 1080) with GROMACS 2018.1 software package [17]. The 2018.1 version has an improved performance because long-ranged non-bonded interactions can be computed on a single GPU. Also, it offers greater control on the usage of the GPUs. The GROMOS 54A7 force field was used to generate the topologies [18]. The version 54A7 was published in 2011 and includes four main improvements: new φ/ψ torsional angle terms were introduced, new atom type for a charged −CH3 in the choline moiety was added, to reproduce free energy of hydration Na^+^ and Cl^−^ ions were modified and additional torsional angles were included. Overall, the GROMOS 54A7 force field has improved stability of secondary structures [18]. Proteins were solvated in a cubic box with SPC water and a minimum distance to the box edge of 1.0 nm [19]. Na^+^ and Cl^−^ were added to neutralize the net charges. For energy minimization the steepest descent minimization algorithm with a step size of 0.01 nm and a maximum of 50,000 steps was applied and then stopped once the maximum force was smaller than 0.1 kJ mol^− 1^ nm^− 1^. For neighbor search, the Verlet cut-off scheme of 1.4 nm was used. To calculate the electrostatic forces, the particle mesh Ewald algorithm with a cut-off of 1.4 nm was applied. For the Van der Waals forces a cut-off of 1.4 nm was adopted. The NVT and NPT equilibration runs took 0.1 ns with 5 × 10^4^ steps and a step size of 2 fs. The production runs were carried out for 100 ns and 10 × 10 ns with 5 × 10^7^ steps with a step size of 2 fs. Temperature and pressure coupling were set to 300 K (velocity rescaling) and 1 bar (Berendsen), respectively. All bonds were constraint to their optimal length using the LINCS algorithm. Energies and coordinates were saved every 10 ps.

### Analysis

#### RMSF

The root-mean-square fluctuation (RMSF) of atomic positions (i.e. standard deviation) gives the displacement of an atom at position **x** with respect to its time-averaged position **x̅**. Cα atoms were used for least-square fitting of the trajectories to the starting structure and RMSF calculations:

1 $$\mathrm{RMSF}\left(\mathbf{x}\right)=\sqrt{\frac{1}{T}\sum\nolimits_{i=1}^T{\left|\left|{\mathbf{x}}_i\left({t}_i\right)-\overline{\mathbf{x}}\right|\right|}^{\mathbf{2}}}$$

where *T* is the total number of time steps within the respective trajectory. To calculate the RMSF, the ten 10 ns long trajectories were combined and treated as if they were a single 100 ns long trajectory.

#### RMSD

The root-mean-square deviation (RSMD) of atomic positions with the BC-loop was calculated after a least-square fit of the C_α_ backbone of the flanking regions. The RMSD is calculated at a time *t* with respect to a given reference structure at time *t*_ref_:

2 $$\mathrm{RMSD}\left({t}_{\mathrm{ref}},t\right)=\sqrt{\frac{1}{N}\sum\nolimits_{i=1}^N{\left|\left|{\mathbf{x}}_i(t)-{\mathbf{x}}_{\boldsymbol{i}}\left({t}_{\mathrm{ref}}\right)\right|\right|}^{\mathbf{2}}}$$

where **x**_*i*_(*t*) is the position of atom *i* at time *t* and *N* is the total number of atoms in that part of the structure to which the RMSD refers, in that case the BC-loop.

#### Clustering

Based on RMSD structures were clustered as described by Daura et al. [20] which consists of the following steps:

1. Define each structure as cluster center
2. Count number of structures within defined cut-off (here 0.2 nm was set) i.e. neighbors
3. Select center with most neighbors, designate it as a cluster and remove set of structures from matrix
4. Repeat until all structures have been assigned to a cluster

Of the biggest 25 clusters the central structures were subjected to non-metric multidimensional scaling to display the structures in a representative two-dimensional space [21]:

1. Choose a random configuration of points in the two-dimensional space
2. Calculate distances between these points
3. Arrange points to maximize rank-order correlation between original RMSD matrix and new space distance
4. Calculate stress and compare to Kruskal’s normalized convergence criterion. If convergence criterion is fulfilled exit, else return to 2.

#### Hydrogen bonds

The hydrogen bonds were determined with GROMACS 2018.1 software package according to the distance and angle of hydrogen donors and acceptors. By default –OH and –NH groups were regarded as donors and –O and –N as acceptors. Hydrogen-donor-acceptor angle and distance cut-offs were set to 30° and 0.35 nm, respectively. Donors and acceptors within that threshold were considered to form hydrogen bonds.

#### Non-bonded interactions

The non-bonded interactions comprise electrostatic and Van der Waals (VdW) interactions. The electrostatic interactions arise from the unequal distribution of charges in molecules and are given by the Coulomb potential E_Coul_. VdW interactions are a combination of dispersion, repulsion and induction forces and are given by the Lennard-Jones (LJ) potential E_LJ_. To compute E_Coul_ and E_LJ_ with Gromacs 2018.1 a simulation rerun of the existing trajectories was invoked. The short-range Coulomb and LJ energies were extracted and summed up.

## Results and discussion

Three different systems, PD-1 unbound, PD-1 – PD-L1 and PD-1 – nivolumab, were each simulated for 100 ns and 10 × 10 ns. Every 10 ps the coordinates of atoms were saved. The structures of PD-1 of each system were extracted and timewise concatenated. RMSF and RMSD were calculated for the BC-loop and the Daura et al. [20] clustering algorithm was performed. Central structures of each cluster, as identified by the Daura algorithm, were then subjected to multidimensional scaling to identify common conformations of the BC-loop of PD-1 across different binding states.

**Fig. 1:**
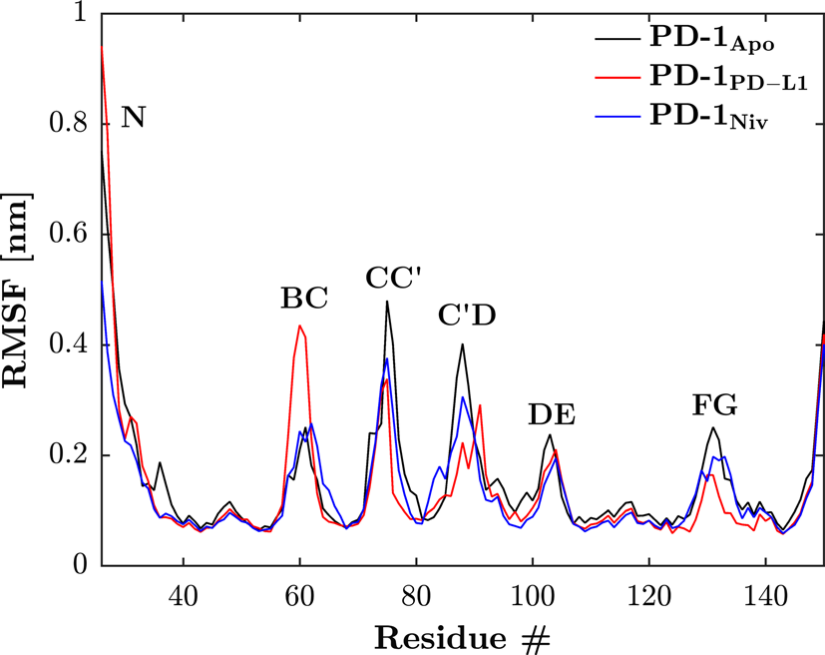
Binding partners influence flexibility of loops. The RMSF of C_α_s of PD-1 unbound (black) and bound to either PD-L1 (red) or nivolumab (blue), when fitted to its respective first frame are shown. Whereas the impact of the binding partners on structured domains is negligible, the RMSF of the loops can change drastically. PD-1_Apo_ has the greatest flexibility except for the BC-loop. The fact that PD-L1 seems to induce more flexibility in the BC-loop led to detailed examination of the BC-loop

The RMSF (Fig. 1) illustrates the influence of the ligands on the flexibility of the PD-1 loops. In β-sheet regions the RMSF of PD-1 (independent of the binding partner) is around 0.1 nm, whereas in areas of loops the values differ considerably. In the CC’-loop, it has been described that PD-1_Apo_ switches between the open and closed conformation whereas PD-L1 shifts the equilibrium towards the closed conformation [11]. Our results confirm these finding as, the RMSF of PD-1_PD-L1_ in the CC’-loop is lower than in PD-1_Apo_. Furthermore, nivolumab binding was found to decrease the flexibility of the N-loop. This is plausible, as it is the only ligand that directly interacts with the N-loop [22]. The C’D-loop could so far only be crystalized when stabilized with pembrolizumab [22]. Here, the C’D-loop was added from the PD-1 – pembrolizumab system (5GGS) to complete the extracellular domain of PD-1. Even though both PD-L1 and nivolumab do not directly interact with the C’D-loop they still decrease the flexibility of the C’D-loop. Except for the BC-loop, the RMSF is highest in PD-1_Apo_ across all domains. Again, PD-L1 does not directly interact with the BC-loop, but whereas PD-L1 still decreases the flexibility in the C’D-loop the opposite occurs in the BC-loop. PD-L1 increases the flexibility of the BC-loop significantly. Therefore, the BC-loop was further examined.

**Fig. 2:**
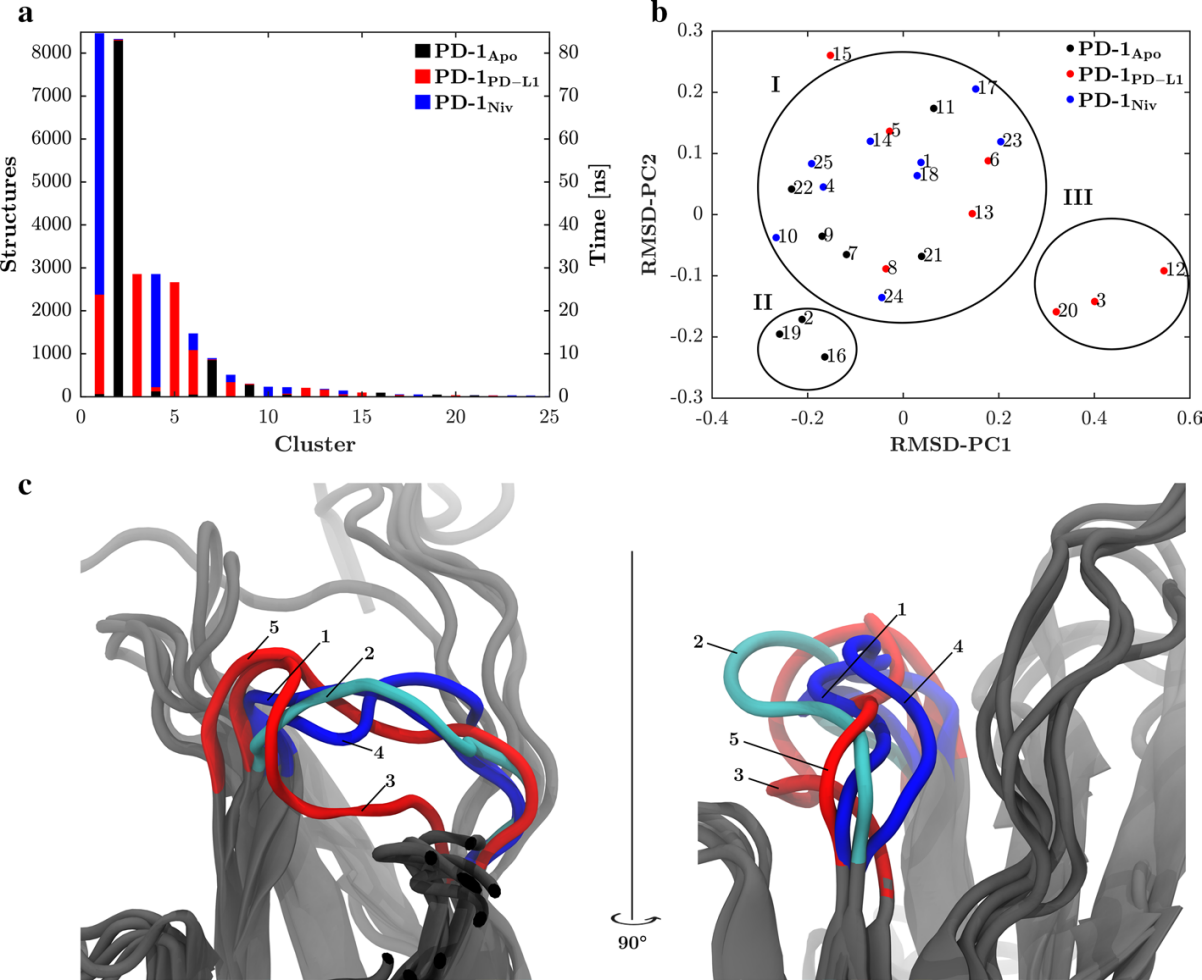
PD-L1 induces a unique BC-loop conformation. The clustering algorithm as described by Daura et al. [20] was applied (**a**). Colors denote the origin of the structures. Only the first 25 clusters are shown. PD-1_PD-L1_ (red) and PD-1_Niv_ (blue) exhibit for 25 and 60% of the time, respectively the same conformation. Over 90% of the structures of PD-1_Apo_ (black) are distributed between cluster 2 and 7. Cluster 3 and 5 contain only PD-1_PD-L1_ structures whereas cluster 4 consist primarily PD-1_Niv_ structures. The number of structures is equivalent to the time the BC-loop resided in a certain conformation. **b** The distance map generated with multidimensional scaling [21] shows the relationship between the conformations found with the Daura et al. [20] clustering algorithm. Clusters with similar structures are in proximity. Three areas are distinguishable (circled by hand) which are named meta-cluster I, II and III. Meta-cluster I is occupied by the PD-1_Apo_, PD-1_PD-L1_ and PD-1_Niv_. Meta-cluster II consists of clusters formed by PD-1_Apo_. Meta-cluster III consists only of structures of PD-1_PD-L1_. This demonstrates a clear shift of the BC-loop conformation in the Nivolumab complex. The colors represent the origins of the central frames. **c** Superimposed crystal structures of the central frames of the first five clusters visualized with VMD 1.9.3. Structures of clusters 1, 4 and 5 exhibit a similar BC-loop conformation. Cluster 2 and 3 exhibit distinct BC-loop conformations. BC-loop is colored; other domains are shown in grey. PD-1_Apo_ structure is shown in cyan instead of black

More than 90% of the structures of PD-1_Apo_ distribute between cluster 2 and 7 in a ratio of 82.5 to 10%, respectively when clustering is based on the RMSD of the BC-loop (Fig. 2a). Two point five percent of the PD-1_Apo_ structures fall into cluster 9. Cluster 1 consists of structures of both, PD-1_PD-L1_ and PD-1_Niv_. Twenty-five and sixty percent of the time the BC-loop of PD-1_PD-L1_ and PD-1_Niv_ exhibit the same conformation, respectively. Clusters 3 and 5 do not show any overlaps and contains only structures of PD-1_PD-L1_. Cluster 4 consists primarily of structures of PD-1_Niv_,he structures of PD-1_Apo_ and PD-1_PD-L1_ are negligible (< 1%). Cluster 6 and 8 show again overlaps and contain structures of PD-1_PD-L1_ as well as PD-1_Niv_. The impact of different cut-offs on clustering is shown in supplementary Figure 1: Larger cut-off allows for more overlaps whereas smaller cutoff yields homogenous clusters, containing configurations from one system only.

The distance map (Fig. 2b) generated with multidimensional scaling [21] shows the relationship between the clusters found with the Daura et al. [20] clustering algorithm. The central frames of each cluster were scaled into the two-dimensional space and clusters of similar structure appear in proximity. Three distinct areas emerged and were named meta-cluster I, II and III. Meta-cluster I consists of clusters of all three systems, whereas meta-cluster II and III only consist of clusters from PD-1_Apo_ and PD-1_PD-L1_, respectively. Eighty percent of the time, the BC-loop of PD-1_Apo_ resides in the meta-cluster II. PD-1_PD-L1_ resides 30% of the time in the meta-cluster III. The BC-loop of PD-1_Apo_, PD-1_PD-L1_ and PD-1_Niv_ reside 20, 70 and 100% of the time in meta-cluster I. The results of the distance map suggest that the BC-loop can exhibit three conformations. Indeed, visualization with VMD 19.3 of the structures of the central frames of the clusters 1 to 5 show three distinct conformations (Fig. 2c). The BC-loop of cluster 1, 4 and 5 (all part of meta-cluster I) appear as bundle which is oriented towards the N-terminal end of PD-1. Cluster 2 (part of meta-cluster II) and cluster 3 (meta-cluster III) show distinct conformations. The BC-loop of the central structure of cluster 3 appears oriented to the center of PD-1. The structures of cluster 2 are in between the bundle of clusters 1, 4, and 5 and cluster 3. Based on the visualization of the central frames it is concluded that multidimensional scaling is a well-suited method to preselect frames to identify conformations with a molecular visualization program.

To investigate convergence behavior, we generated clusters not only for the whole 100 ns trajectory but for increasing parts of it, i.e. the first 10 ns, 20 ns, 30 ns etc., with a constant cut-off of 0.2 nm. As the simulation samples increasing percentage of phase space, the numbers of clusters increase (Supplement Figure 2). Around 70 ns the number of clusters of the BC-loop of the PD-1_Apo_ simulation seems to level off at 18 clusters. This indicates sufficient sampling over configuration space and that the system reaches convergence within 100 ns.

**Fig. 3:**
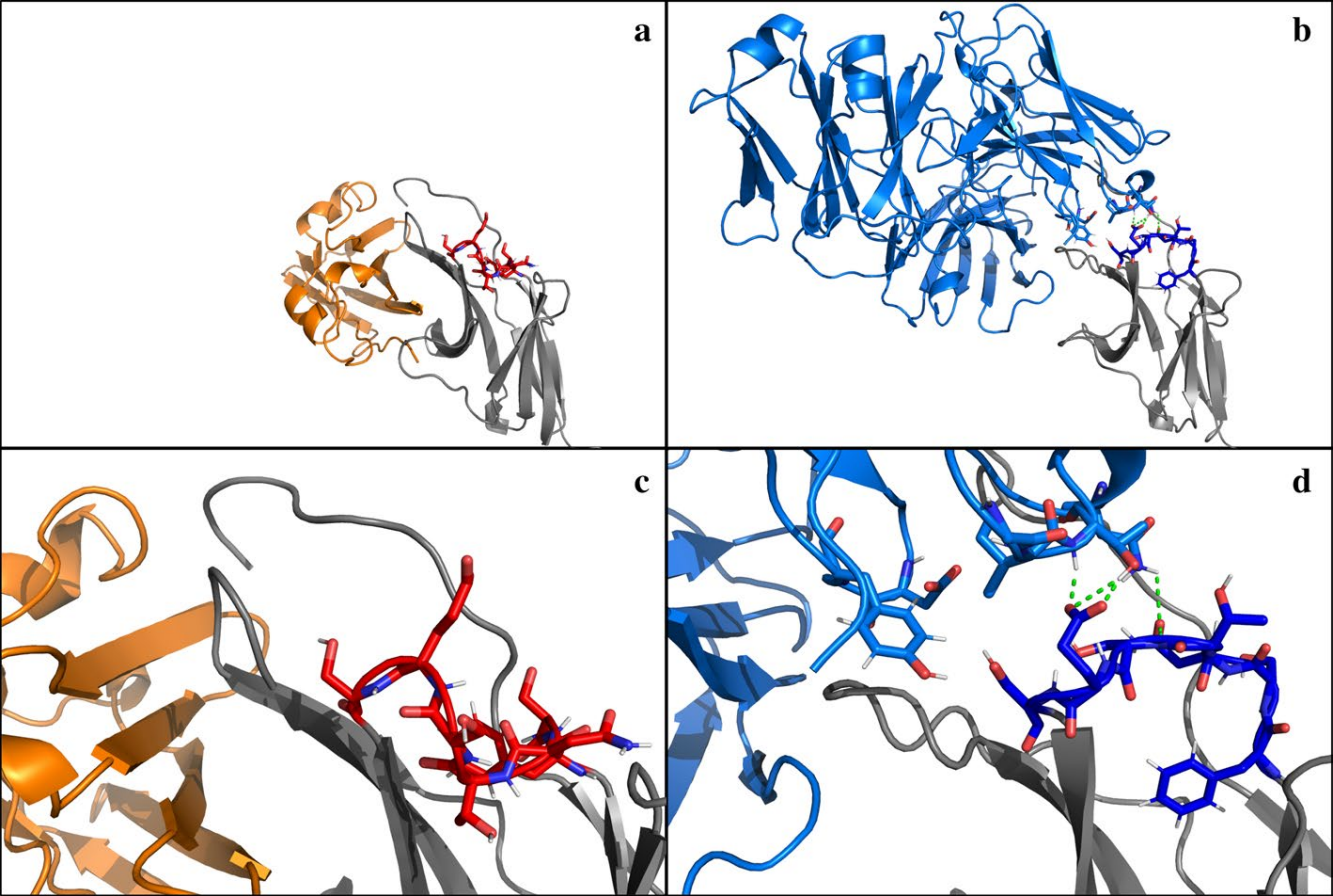
Binding of PD-L1 and Nivolumab to PD-1. **a** Central member structure of cluster 3 of the BC-loop, binding to PD-L1; PD1- in grey, with the BC-loop in red sticks and PD-L1 in orange. **b** Central member structure of cluster 1 of the BC-loop, binding to Nivolumab; PD-1 in grey, with the BC-loop in blue sticks and Nivolumab in light blue. Hydrogen bonds between PD-1 and Nivolumab observed in this structure indicated in green. **c**, **d** zoomed in views of (**a**, **b**), respectively. PD-1 is in the same orientation in all four panels

Furthermore, the results suggest a complex mechanism of conformational changes of the BC-loop. Depending on the binding partner the conformational change of the BC-loop is based on the conformational selection or the induced fit model [23]. Both concepts assume a conformational change of the unbound (apo-)protein to bind a ligand. The fundamental difference is whether the conformational change happens before or after ligand binding. In the induced-fit model the partner induces the active binding conformation of the protein. On the hand, in the conformational-selection model the active and inactive forms coexist even in absence of a ligand, but the ligand shifts the equilibrium to the active conformation [24]. The BC-loop exists in PD-1_Apo_ to 20% in conformation I and to 80% in conformation II. Upon binding to nivolumab the equilibrium is shifted to 100% of conformation I. PD-L1, on the other hand, induces a BC-loop conformation which is not exhibited in PD-1_Apo_. Analysis of the PD-1_PD-L1_ trajectory has shown that after 70 ns the BC-loop switches to conformation III (Fig. 3).

**Fig. 4:**
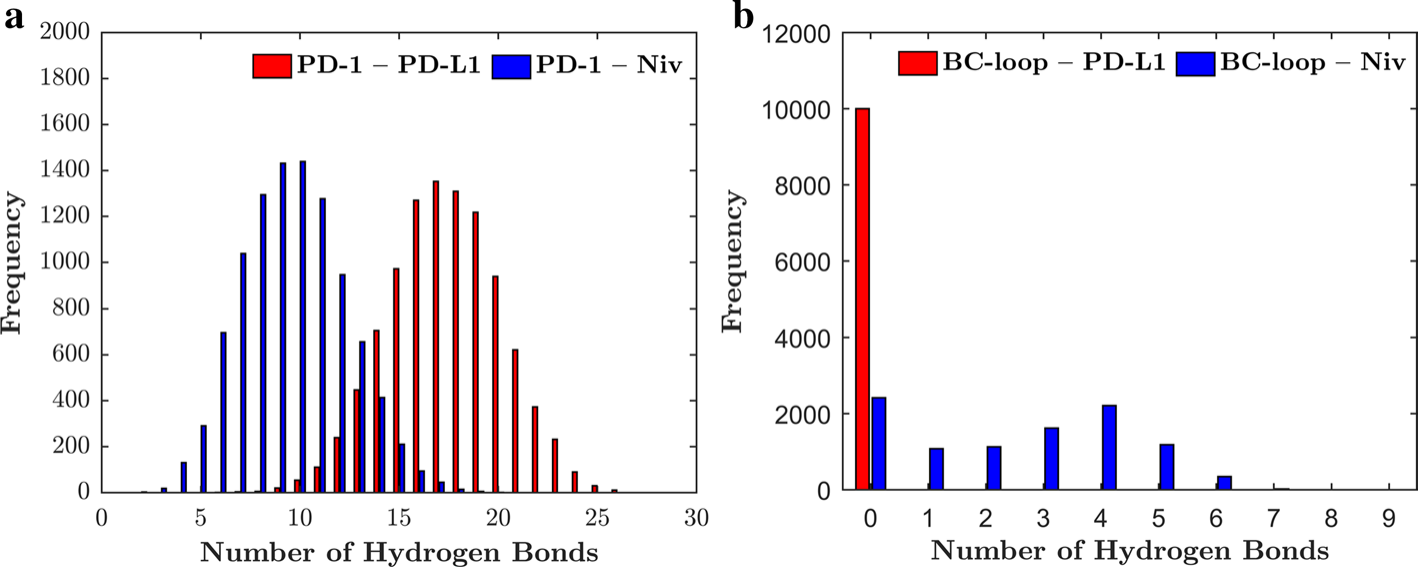
The BC-loop does not form any hydrogen bonds with PD-L1. **a** The frequencies of the number of hydrogen bonds formed between PD-1 and PD-L1 (red) and PD-1 and nivolumab (blue) over the course of 100 ns are shown. The PD-1 – PD-L1 complex has a peak of 17 formed hydrogen bonds which occur in 1400 frames (which corresponds to 14 ns). The PD-1 – Nivolumab complex formed 10 hydrogen bonds for 1500 frames. **b** Between the BC-loop and PD-L1 no hydrogen bonds are formed. The BC-loop and nivolumab form 2200 times 4 hydrogen bonds over the course of 100 ns. Also, for 2300 frames no hydrogen bond occurs between the BC-loop and nivolumab

**Fig. 5:**
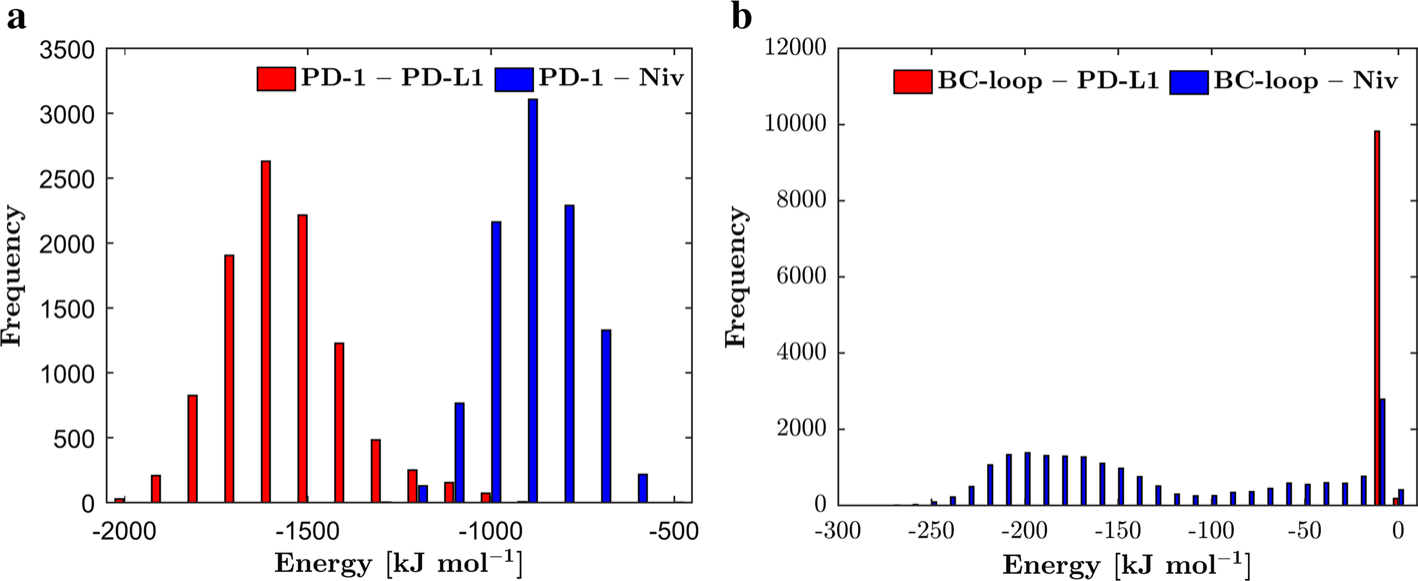
No non-bonded interactions between the BC-loop and PD-L1. **a** The non-bonded interaction energies between PD-1 and PD-L1 (red) and PD-1 and nivolumab (blue) are shown as a histogram and are grouped in packages of 100 kJ mol^− 1^. The non-bonded interaction energy between PD-1 and PD-L1 ranges from − 1000 to − 2000 kJ mol^− 1^ with a peak of 2700 frames at − 1600 kJ mol^− 1^. The energy distribution between PD-1 and nivolumab ranges from − 500 to − 1200 kJ mol^− 1^. The peak is at − 900 kJ mol^− 1^ with 3100 occurrences. **b** The non-interaction energies between the BC-loop and two ligands is grouped into packages of 10 kJ mol^− 1^. The energy between the BC-loop and PD-L1 peaks at-10 kJ mol^− 1^. This peak occurs due to the minor distortions (between 0 and − 1 kJ mol^− 1^). The non-bonded interaction energy between the BC-loop and nivolumab ranges from 0 to − 250 kJ mol^− 1^

Hydrogen bond and non-bonded interaction energy analyses were done to examine potential reasons for the conformational change of the BC-loop upon PD-L1 binding and are shown as histograms (Figs. 4 and 5). Overall PD-1 forms more hydrogen bonds with PD-L1 (peak at 17 hydrogen bonds) than nivolumab (10 hydrogen bonds) over the course of 100 ns (Fig. 4a). However, no hydrogen bonds are formed between the BC-loop and PD-L1 (Fig. 4b). Nivolumab on the other hand forms up to 6 hydrogen bonds with the BC-loop during 100 ns. Coherently, the non-bonded interaction energies show a similar pattern. The BC-loop does not interact with PD-L1 via VdW or electrostatic forces. The non-bonded interaction energy between the BC-loop and nivolumab peaks at − 120 kJ mol^− 1^. The results corroborate with the fact that no interactions between the BC-loop and PD-L1 have been mentioned in the literature so far. Indeed, visualization of the central frame of cluster 3 (Fig. 3a and c) verify that interaction between the BC-loop and PD-L1 is spatial not possible. For nivolumab on the hand evidence of such interaction have been reported. Again, visualization of the central frame of cluster 1 shows that hydrogen bonds between the BC-loop and nivolumab are formed (Fig. 3b and d).

In a crystallization experiment Lee et al. [25] concluded (referring to Zak et al. [10]) that nivolumab induces a conformation in the BC-loop which is incompatible with PD-L1. Based on the results of the MD simulation we cannot confirm this. Admittedly, nivolumab induces a conformational change in the BC-loop however, this conformation is compatible with PD-L1. In fact, the BC-loop shares this conformation when bound to PD-L1 for 25% of the time. In another crystallization experiment it has been described that the BC-loop is shifted ~ 5.3 Å away when it binds to nivolumab compared to PD-L1 [22]. These findings could be confirmed as indeed, 30% of the time the BC-loop of PD-1_PD-L1_ is shifted away from the BC-loop of PD-1_Niv_. As hydrogen bonds and non-bonded interactions are not the driver for the conformational changes, we assume entropic causes. To investigate this we suggest free energy calculations [10].

## Conclusion

The immune checkpoint receptor PD-1 has been identified as a key target in cancer immunotherapy. The PD-1 blocking antibody nivolumab which inhibits the PD-L1 binding was recently approved by the FDA. Even though PD-1 is already used as a drug target the exact mechanism of the receptor is still unknown. Here we present the results of the first MD simulations of PD-1 with a complete extracellular domain and a focus on the role of the BC-loop of PD-1 upon binding PD-L1 or nivolumab. Visualization of the structures proofs that the combination of Daura et al. [20] clustering and multidimensional scaling is a valid approach to identify conformations. The BC-loop of PD-1 can exhibit three conformations and occurrence of the conformations depends on the binding partner. Furthermore, we identified that upon nivolumab binding the BC-loop changes the conformation as described in the conformational selection model. PD-L1, on the other hand, does not even directly interact with the BC-loop but it nonetheless induces a conformational change in the BC-loop. This allosteric effect could play a crucial role in the activation of the PD-1 receptor.

In the future we will search for movement patterns of PD-1 based on unsupervised numerical methods and evidence from the literature.

## Supplementary information

**Additional file 1: Supplement Figure 1.** Comparison of cut-offs. For the clustering algorithm it is necessary to define a cut-off (in nm). Structures within the cutoff are seen as similar and are grouped. On the one hand the cut-off must be small enough to distinguish between conformations structurally different. On the other hand, it must not bet too small to avoid over-differentiation. (**A**) When the cut-off is set to 0.3 nm five clusters are found. (**B**) 25 clusters are found when the cut-off is set to 0.2 nm. (**C**) Over 100 clusters are found with a cut-off of 0.1 nm.

**Additional file 2: Supplement Figure 2.** Incremental clustering indicates convergence of the simulation. Clustering was performed for the PD-1_Apo_ simulation with a constant cut-off of 0.2 nm for sub-trajectories of the first 10 ns, 20 ns etc., see horizontal axis (time). With increasing length of sub-trajectory the number of clusters increases (vertical axis) until it finally levels off at 70 ns. This indicates sufficient sampling allover configuration space and convergence of the simulation.

## Data Availability

The datasets used and/or analysed during the current study are available from the corresponding author on reasonable request.

## Abbreviations

APC: Antigen-presenting cells
cHl: Classical Hodgkin lymphoma
LJ: Lennard-Jones
MD: Molecular dynamics
MHC: Major histocompatibility complex
NSCLC: Non-small-cell lung carcinoma
PD-1: Programmed cell death protein I
PD-L1: Programmed cell death protein I ligand I
RMSD: Root-mean-square deviation
RMSF: Root-mean-square fluctuation
TCR: T cell receptor
VdW: Van der Waals
VSC: Vienna Scientific Cluster
