# Supplementary figures and images for: Molecular dynamics of the immune checkpoint programmed cell death protein I, PD-1: conformational changes of the BC-loop upon binding of the ligand PD-L1 and the monoclonal antibody nivolumab
Source: BMC Bioinformatics. 2020 Dec 14;21(Suppl 17):557. doi: 10.1186/s12859-020-03904-9 (PMC7734776; doi:10.1186/s12859-020-03904-9)

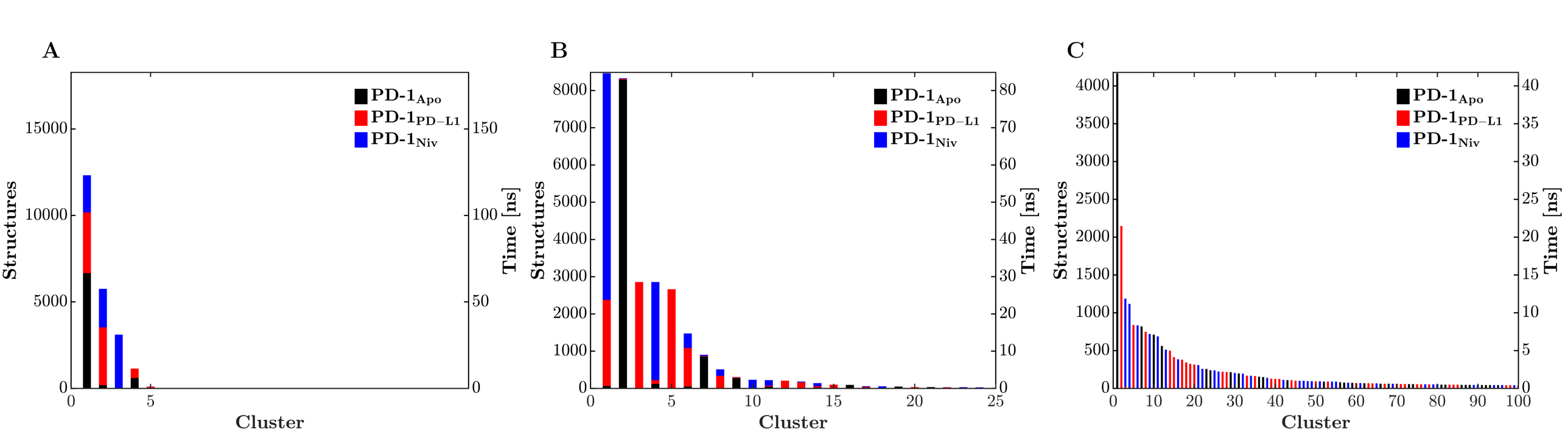

Supplement: Supplementary file 1 — Additional file 1: Supplement Figure 1. Comparison of cut-offs. For the clustering algorithm it is necessary to define a cut-off (in nm). Structures within the cutoff are seen as similar and are grouped. On the one hand the cut-off must be small enough to distinguish between conformations structurally different. On the other hand, it must not bet too small to avoid over-differentiation. (A) When the cut-off is set to 0.3 nm five clusters are found. (B) 25 clusters are found when the cut-off is set to 0.2 nm. (C) Over 100 clusters are found with a cut-off of 0.1 nm. [file 12859_2020_3904_MOESM1_ESM.png]

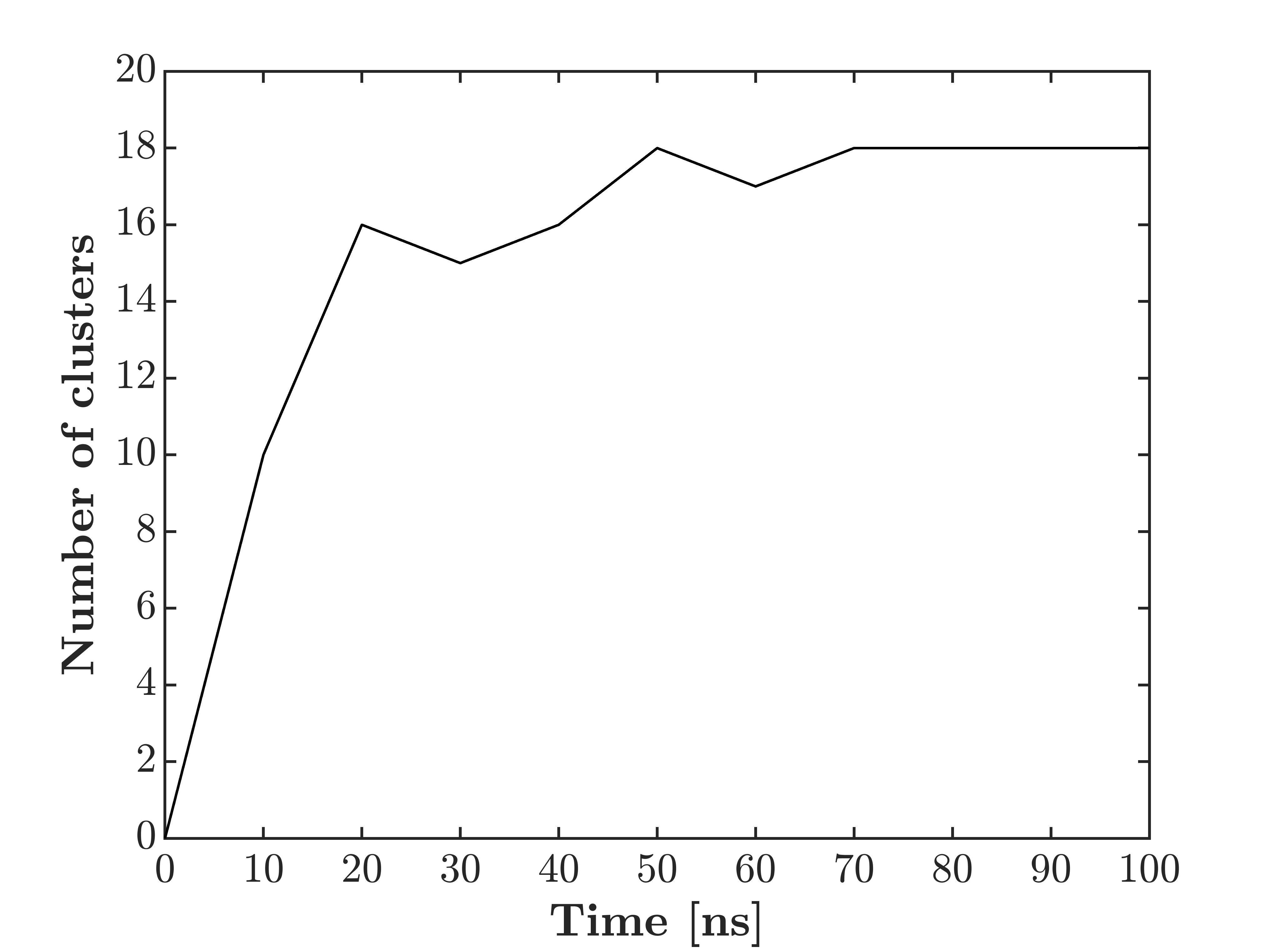

Supplement: Supplementary file 2 — Additional file 2: Supplement Figure 2. Incremental clustering indicates convergence of the simulation. Clustering was performed for the PD-1Apo simulation with a constant cut-off of 0.2 nm for sub-trajectories of the first 10 ns, 20 ns etc., see horizontal axis (time). With increasing length of sub-trajectory the number of clusters increases (vertical axis) until it finally levels off at 70 ns. This indicates sufficient sampling allover configuration space and convergence of the simulation. [file 12859_2020_3904_MOESM2_ESM.png]
